# Progress in Medicine

**Published:** 1899-02-25

**Authors:** 


### Progress in Medicine.

### THE ALIMENTARY TRACT.

(*Continued from page* 349.)

**Intestines.**—Ulcers of the duodenum resulting in perforation and peritonitis although seldom diagnosed previous to operation are not uncommon. Wanach[1] reports a case successfully operated upon, and reviews the cases already reported. The localisation of the site of perforation is difficult, the condition frequently being mistaken for perforation of the vermiform appendix, for the pain and tenderness is generally greatest on the right side. If the conditions have followed appendicitis it will generally be found that pressure over the inflamed appendix will elicit tenderness in that region, and that pain is referred to the epigastrium, signs not present in duodenal perforation. The clinical history of the onset and course of the acute trouble will afford valuable aid in localising the disorder. The commonest site of perforation is at the apex of the curvature of the second part of the duodenum, above the point of entry of the pancreatic and bile ducts. There is generally an indurated and thickened area with a punched out centre. Surgical treatment consists in either inverting and suturing the edges, or excising the ulcer and bringing the cut surfaces into apposition. The abdominal wound is treated by packing and drainage. Malignant disease of the duodenum is not common, being recorded only seven times out of forty-two thousand autopsies according to Schlesinger.[2] In a case under his own care the second part of the duodenum was affected, there being jaundice and repeated vomiting. The lumen of the bowel was not narrowed. Mauclaire and Durrieux[3] describe a case where the carcinomatous growth almost encircled the bowel just at the point of entry of the bile duct. There was no jaundice, and although during life there were signs of acute obstruction, no dilatation of stomach or duodenum was found at the post-mortem examination. No tumour could be felt during life. Hypertrophic tuberculosis of the colon[4] may lead to intestinal obstruction. The symptoms closely resemble those of syphilitic disease or new growth. The mucous membrane is invaded by a diffuse growth of granulation tissue, which may replace all the coats and spread to neighbouring cellular tissue. Microscopical examination proves the growth to be of tubercular origin, but the demonstratum of bacilli is a matter of some difficulty. The points attacked most frequently are the two ends of the colon, the cæcum, and the rectum. A peculiar condition of the colon in pernicious anæmia was described at the meeting of the Pathological Society by Fletcher.[5] The mucous membrane was rough and warty, leathery in consistence, and under the microscope showed necrotic changes. There were old-standing fibrosis of the submucous tissue. The question arose as to whether the condition was concerned in the causation of the disease or was due to arsenic, which had been administered in small quantities.

**The Liver.**—Several interesting physiological points were detailed in the discussion on the secretion of bile in man at the physiological section of the British Medical Association.[6] Bain showed that there is no relation between the weight of the individual and the amount of bile secreted. The influence of the central nervous system was evidenced by the jaundice which occasionally follows fright. The quantity of bile depends to some extent upon the quantity of fluid ingested and the amount of exercise taken. Bile and bile salts administered by the mouth undoubtedly acted as cholagogues. Iridin, too, appeared to be a drug of some power. Rutherford had watched the secretion in a case of biliary fistula under his care. High secretion followed food ingestion, and continued for some hours. Morphia or alcohol had no effect upon the flaw, but the fluids and salts of the bile were increased by salicylate of soda.

**Cirrhosis of Liver.**[7]—Adami points out that alcohol leads rather to fatty than to progressive cirrhotic change in the liver, which is in his opinion an infective process associated with bacterial growth. Both in progressive cirrhosis in man and in the disease known as the Pictou disease in cattle, in which fibrotic liver changes are very marked, there is to be found in the newly-formed connective tissue of the liver a diplococcus or ovoid bacterium. When isolated, the organism is polymorphic, is pathogenic to animals, and may again be recovered from the tissue of the liver. Further investigations have convinced Adami that the organism described was a variety of the colon bacillus, and whilst not claiming that all forms of fibrosis are due to this cause, yet he maintains there is an undoubted relationship between the presence of these organisms and the development of ordinary cirrhosis. The production of cirrhosis of the liver by the introduction of micro-organisms or their toxins into the system has been established by the experimental work of Krawkow, Seagliosi, and others.[8] The microbes used were staphylococci, B. pyocyaneus, subtilis, and septicus putridus. In all cases there were changes in the cells of a necrotic character such as are found in the form of cirrhosis associated with gastro-intestinal disturbances in young children, apart from syphilis and other causes. This variety is probably due to the absorption of toxic substances from the bowel. The French school maintain that the consumption of alcoholic beverages plays a great part in the causation of hepatic fibrosis. Saingery holds that whilst alcohol produces steatosis, wine produces cirrhosis. According to Lancereaux, the special constituent of noxious character in wines is the salts of potash which are added in the process known as "plastering." The matter cannot be regarded as decided, but it is noteworthy that attempts to produce cirrhosis by the administration of pure alcohol have been singularly unsuccessful. Another factor in the causation of cirrhosis is obstruction of the bile ducts by gall-stones, tumours, &c. A case of this kind is reported by Workman,[9] and it is interesting in this connection to note that Harley and Barrett[10] have obtained by ligature of the hepatic ducts in animals a marked increase of interlobular tissue. A case of acute atrophy of the liver is described by O'Neill[11] in a man aged 46. Causation was obscure. There was jaundice, headache, sickness, and intense pain over the liver, bowels constipated, urine scanty.

The pulse was good and temperature normal until death, on the fifth day, when it reached 104 deg. Fahr. Towards the end there was delirium, drowsiness, and coma. The liver was smaller and the spleen larger than in health. The disease was a general infection, probably of microbic origin. In marked contrast with the foregoing is a case recorded by Daly.[12] A woman, aged 26, was suddenly seized with faintness and vomiting. On the following day there was jaundice, and some enlargement of the liver; temperature, 102; pulse, 130. The patient passed into a typhoid condition, there was suppression of urine, and death resulted on the third day. As there was no diminution in the size of the liver, and no leucin or tyrosin in the urine, acute yellow atrophy was excluded, and the case designated one of icterus gravis, probably due to micro-organisms.

**Gall Bladder.**—Richardson[13] records ten cases of acute inflammation of the gall bladder of sudden onset in persons of apparently perfect health. Acute infectious disease and prolonged cholelithiasis could be excluded. The condition is a very serious one, as rupture or gangrene may result. Surgical intervention is almost always required. Directly or indirectly these cases are generally the result of gall stones, but in four of the cases no gall stone was detected. The irritation set up in the gall bladder by the presence of stones excites inflammation and opens a path of infection. It appears probable that infection is from the blood rather than from the common bile duct or the adjacent bowel. Distension of the gall bladder is an important factor in acute cholecystitis, and experiment has shown that obstruction in the biliary passages favours rather than hinders an ascending infection. The organisms found are pneumococcus, B. coli, and the typhoid bacillus. Paroxysmal pain in the right half of the abdomen, tenderness, vomiting, abdominal distension, fever, signs of acute obstruction, and peritonitis are the general symptoms. The distended gall bladder can sometimes be felt in its normal position. Treatment consists in so draining that the viscus does not infect the surrounding peritoneum. Stevens advocates early exploratory incision in cases where obscure digestive troubles and slight icterus suggest the possibility of disease of the gall bladder. He notes certain reflex symptoms often excited by gall-bladder disease, namely, palpitation of the heart, pain in the urinary bladder, and spasmodic spells of coughing. Wendel's[14] contribution to the symptomatology and diagnosis of cholelithiasis in infancy and in childhood is founded on sixteen cases occurring in persons under eight years of age. Each case was corroborated by detecting the calculi either in the stools, or on the operating table, or at the autopsy. A predisposing cause is the application of a tight abdominal binder to the infant during the early months of life. Many of the painful gastro-intestinal disorders of children are due to passage of gall stones, which, although it may not give rise to jaundice, may often lead to changes in the urine. If the duct be totally obstructed the symptoms are characteristic, and the temperature rises to 100 or 101 deg. Fahr. In cholecystitis there is generally vomiting and eclampsia, with tenderness over the gall bladder. In two of Wendel's cases perforation of the gall bladder and general peritonitis resulted. In four others no inconvenience was detected during life, but stones were found in the gall bladder after death. The pain is generally referred to the epigastrium, is generally accompanied by vomiting, and followed by local tenderness, with jerky costal breathing. Jaundice in children, caused by gall stones without pain, is rare. Acholic fæces may be of green colour. The treatment of hepatic colic by olive oil is strongly advocated by Barth.[15] Given during an attack, its lubricating action sometimes affords immediate relief. From the duodenum it enters the bile duct, and may facilitate the escape of the impacted stone. Whether the oil can dissolve the stone is uncertain, but Brockbank, using a bath of oil at body temperature, has seen a calculus of 1·6 grams lose 1·21 grams in weight in four days. The oil in the gut is partly converted into fatty acids and glycerine, and the passage of these bodies through the liver excites a copious flow of bile, which not only favours the progress of the calculus towards the intestine, but also expels mucus, epithelial masses, and inspissated bile that may be in the passages. Dose, 150 grams before breakfast. Gautier[16] has tried ox bile internally in biliary colic. It is first decolorised and then sterilised at 104 deg. or 105 deg. C. Three grams were given twice daily after food. The results were most successful, both in aiding the expulsion and in preventing the formation of calculi. He recommends this treatment after operations for the evacuation of calculi.

[1] Archiv. für Klinische Chirurg., vol. lvi., No. 2. [2] Wiener Klin. Woch., 1898, No. 10, p. 245. [3] Bull. Soc. Anat., Paris, April, 1898. [4] Presse Médicale, May 18, 1898. [5] Epit. Brit. Med. Jour., Dec. 24, 1898. [6] Brit. Med. Jour., Sept. 17, 1898. [7] Brit. Med. Jour., Aug. 13, 1898, and Oct. 22. [8] Internat. Med. Mag., March, 1898. [9] Glasgow Med. Jour., March, 1898. [10] Brit. Med. Jour., Dec., 1898. [11] Lancet, April 30, 1898. [12] Ibid. [13] Amer. Jour. of Med. Sci., June 1898. [14] Med. Rec., July 9, 1898. [15] La Semaine Médicale, No. 56, p. 441. [16] Epit. 177 Brit. Med. Jour., Sept. 3, 1898.

